# Dissecting dynamic plant virus synergism in mixed infections of poleroviruses, umbraviruses, and tombusvirus-like associated RNAs

**DOI:** 10.3389/fmicb.2023.1223265

**Published:** 2023-07-06

**Authors:** Anna Erickson, Bryce W. Falk

**Affiliations:** Department of Plant Pathology, University of California, Davis, Davis, CA, United States

**Keywords:** co-infection, transcomplementation, polerovirus, umbravirus, tlaRNA

## Abstract

Mixed infections of a plant infecting polerovirus, umbravirus, and/or tombusvirus-like associated RNAs (tlaRNAs) produce unique virus disease complexes that exemplify “helper-dependence” interactions, a type of viral synergism that occurs when a “dependent” virus that lacks genes encoding for certain protein products necessary for it to complete its infection cycle can utilize complementary proteins encoded by a co-infecting “helper” virus. While much research has focused on polerovirus-umbravirus or polerovirus-tlaRNA interactions, only recently have umbravirus-tlaRNA interactions begun to be explored. To expand on the limited understanding of umbravirus-tlaRNA interactions in such disease complexes, we established various co-infection pairings of the polerovirus turnip yellows virus (TuYV), the umbravirus carrot mottle virus (CMoV), and three different tlaRNAs—carrot red leaf virus aRNAs (CRLVaRNAs) gamma and sigma, and the TuYVaRNA ST9—in the model plant *Nicotiana benthamiana*, then investigated the effects of these different co-infections on tlaRNA systemic movement within the host, and on virus accumulation, and aphid and mechanical transmission of each of these viruses. We found that CMoV alone could support systemic movement of each of the tlaRNAs, making this the second report to demonstrate such an interaction between an umbravirus and tlaRNAs. We also report for the first time that CMoV could also impart mechanical transmissibility to the tlaRNAs sigma and ST9, and that co-infections of either of these tlaRNAs with both TuYV and CMoV increased the efficiency with which TuYV could be mechanically co-transmitted with CMoV.

## Introduction

Helper-dependence, also known as transcomplementation, is a particularly interesting example of virus synergism between co-infecting viruses. In such infections, the “helper” virus encodes a gene(s) product that the “dependent” virus lacks but can utilize, and in some instances this interaction is obligatory for the dependent virus to complete its infection cycle as these proteins facilitate within host movement and/or transmission between hosts (Rochow, 1972; Latham and Wilson, 2008; Syller, 2012; Alcaide et al., 2020). In many instances, such co-infections also result in significantly enhanced symptom development in the host and significantly increased accumulation of one or more of the co-infecting viruses (Murant et al., 1988; Barker, 1989; Murant, 1990; Passmore et al., 1993; Sanger et al., 1994; Li et al., 2017; Zhou et al., 2017). Mixed infections comprised of a polerovirus, an umbravirus, and/or a co-infecting tombusvirus-like associated RNA (tlaRNA) exemplify this type of viral synergism.

Multiple disease complexes comprised of a polerovirus, an umbravirus, and/or a satellite virus or tlaRNA have been identified that are responsible for causing severe disease outbreaks in economically important crops (Watson and Serjeant, 1964; Falk and Duffus, 1984; Passmore et al., 1993; Sanger et al., 1994). The groundnut rosette disease (GRD) complex, caused by co-infection of the polerovirus groundnut rosette assistor virus (GRAV), the umbravirus groundnut rosette virus (GRV), and either one of two associated satellite RNAs—which appear to be responsible for symptom severity and GRV encapsidation by GRAV produced capsids—is perhaps the most well studied example of such a viral disease complex that has caused devastating losses in crop production (Storey and Ryland, 1955; Hull and Adams, 1968; Murant et al., 1988; Murant, 1990; Taliansky et al., 1996; Naidu et al., 1998; Taliansky et al., 2000). Another notable example is the carrot motley dwarf (CMD) disease complex, comprised of the polerovirus carrot red leaf virus (CRLV), the umbravirus carrot mottle virus (CMoV), and/or one of several CRLV associated RNAs (CRLVaRNA; Watson et al., 1964, 1998; Murant et al., 1969). CMD occurs globally, anywhere that carrots are produced, and has caused epidemics resulting in severe crop losses (Watson and Serjeant, 1964; Watson and Falk, 1994).

Viruses in the genus *Polerovirus* (family *Solemoviridae*) are phloem limited and obligately vectored by aphids, often in a species-specific manner; they encode their own capsid proteins (CP) and are capable of *in planta* systemic movement (Brault et al., 1995; Mayo and Ziegler-Graff, 1996; Peter et al., 2009; Pagán and Holmes, 2010). Umbraviruses are mechanically transmissible, non-phloem limited viruses in the family *Tombusviridae* that can move cell-to-cell and systemically within their plant hosts but lack genes encoding for their own CP and the capacity to be independently transmitted by an insect vector (Nurkiyanova et al., 2001; Taliansky et al., 2003; Taliansky and Robinson, 2003; Kim et al., 2007). TlaRNAs are small (~2.8 kb) putative viruses that only encode a replicase protein, making them incapable of independent movement within their plant hosts or transmission to new hosts, and as such are exclusively found co-infecting with a polerovirus, sometimes in association with an umbravirus (Falk and Duffus, 1984; Passmore et al., 1993; Watson et al., 1998; Campbell et al., 2020).

**Figure 1:**
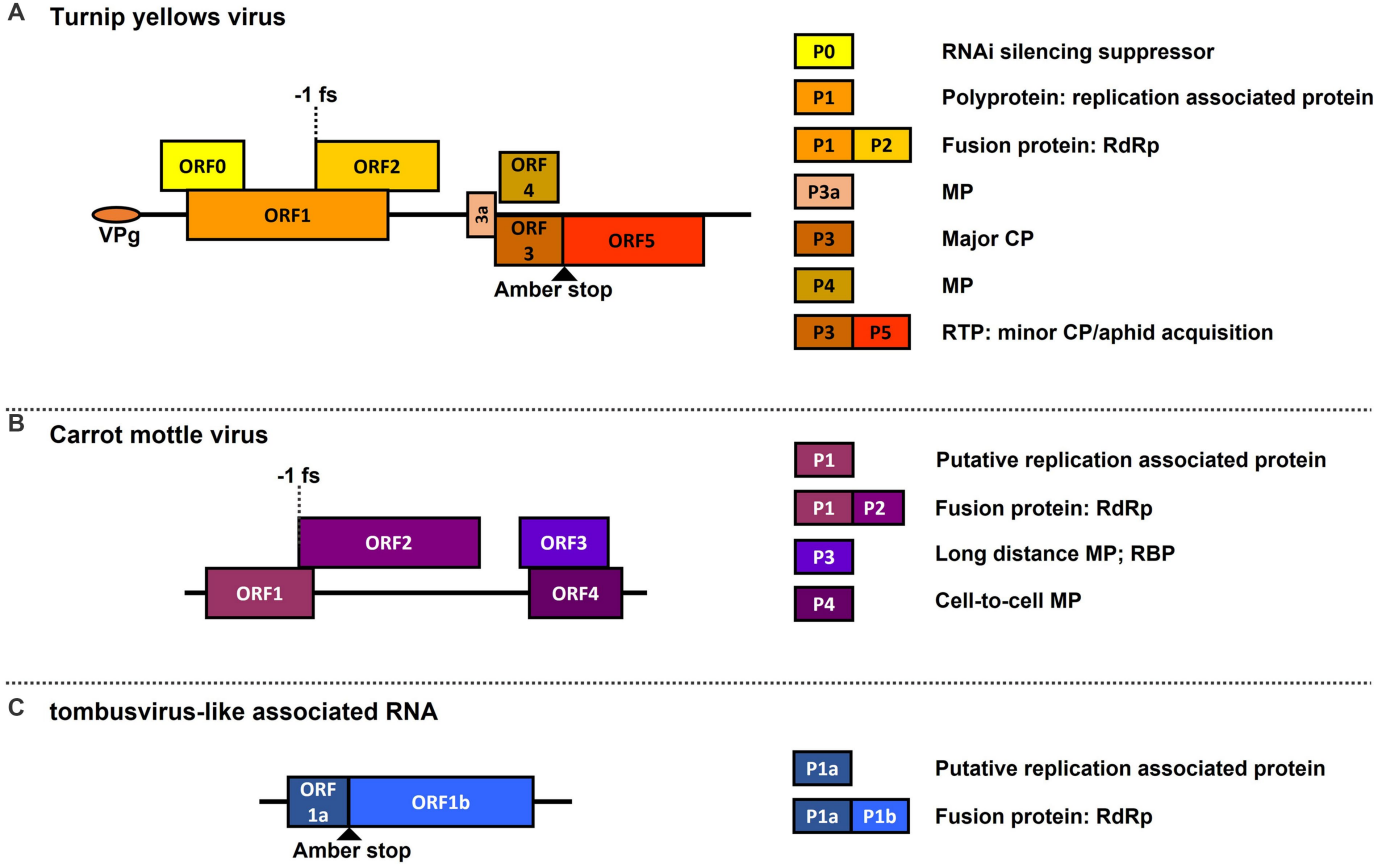
Genome maps of viruses used in this study. Depicted are graphical representations of: **(A)** the polerovirus turnip yellows virus (TuYV); **(B)** the umbravirus carrot mottle virus (CMoV); and **(C)** a general representation of tombusvirus-like associated RNA (tlaRNA) genome structure. Diagrams are drawn to scale. Solid black lines depict the length of the viral RNA genome. Boxes indicated the open reading frames (ORFs) encoded by each virus, and their positions in relation to the solid black line indicate in which reading frame register (RFR) they occur (Above the line: RFR1; on the line: RFR2; beneath the line: RFR3). Key translation features such as amber stop codons used for translational readthrough and −1 frameshift sites are depicted. The boxes to the right of the genome indicate the known functions of proteins encoded by the corresponding ORFs depicted in the viral genomes. RNAi, RNA interference; RdRp, viral replicase protein; MP, movement protein; CP, capsid protein: RTP, readthrough protein; RBP, RNA binding protein.

One such virus complex is the polerovirus TuYV with the ST9aRNA in shepherd’s purse [*Capsella bursa-pastoris* (*C. b-p*)] plants, which produces significantly enhanced symptom development and TuYV accumulation, and in which the ST9aRNA gains systemic movement and aphid transmissibility through encapsidation by TuYV capsid proteins (Falk and Duffus, 1984; Passmore et al., 1993; Sanger et al., 1994). Another pertinent example is the aforementioned carrot motley dwarf (CMD) disease complex, which also shows enhanced symptom development in various apiaceous hosts, and in which CMoV and CRLVaRNAs are known to be dependent on CRLV for aphid transmission (Murant et al., 1969; Waterhouse and Murant, 1983; Murant et al., 1985; Watson and Falk, 1994; Watson et al., 1998). Genome maps of each of these viruses and the proteins they encode are depicted in Figure 1. It has also been found that some poleroviruses can, presumably, utilize the cell-to-cell movement proteins (MPs) of a co-infecting umbravirus, allowing the polerovirus to presumably escape phloem limitation and become mechanically transmissible (Hoffman et al., 2001; Ryabov et al., 2001a; Zhou et al., 2017). While there are a good number of studies on polerovirus-tlaRNA and polerovirus-umbravirus interactions, there currently exist few studies on umbravirus-tlaRNA interactions.

To further parse the various virus-virus interactions that occur in these disease complexes, we used aphid inoculation of TuYV in combination with agroinfiltration of infectious clones of CMoV and three tlaRNAs—CRLVaRNAs Gamma and Sigma, and ST9aRNA, heretofore referred to simply as Gamma, Sigma, and ST9—to generate single, double, and triple infections of each of these viruses and examine their effects on symptom development, systemic movement of tlaRNAs, and on aphid and mechanical transmission of each of these viruses. In this study, TuYV was used in place of CRLV because we did not have on hand the specific aphid vector (*Cavariella aegopodii*) of this virus (Murant et al., 1969; Elnagar and Murant, 1978), whereas we did have the aphid vector (*Myzus persicae*) of TuYV as well as an active culture of TuYV maintained in *C. b-p.* plants. Our results show that CMoV appears to be a driver of symptom development when co-infected with TuYV and/or Sigma or ST9. CMoV also facilitated the systemic movement of all three tlaRNAs—albeit with differing efficiencies—in the absence of TuYV, corroborating the results of a recent study that demonstrated umbravirus-facilitated tlaRNA systemic movement in the tobacco bushy top disease (TBTD) complex (Chen et al., 2022).

CMoV could also facilitate mechanical transmission of Sigma and ST9 (but not Gamma) and TuYV; the transmission rate of TuYV from plants co-infected with only CMoV was very low, however when co-infected with both CMoV and either Sigma or ST9, the transmission rate of TuYV greatly increased. This is the first report to demonstrate that umbravirus co-infection can impart mechanical transmissibility to tlaRNAs and that some tlaRNAs appear to increase the efficiency with which a co-infecting polerovirus can be mechanically transmitted from plants also co-infected with an umbravirus. Effects of co-infection on the ability of CMoV and the tlaRNAs to be co-aphid transmitted with TuYV, as well as effects on the accumulation of each of these viruses were also determined.

## Materials and methods

### Establishing mixed infections

Combined aphid and *Agrobacterium tumefaciens*-mediated inoculations were used to generate the various single, double, and triple infections examined in this study which are listed in Table 1. For TuYV inoculation, non-viruliferous green peach aphids (*Myzus persicae*) were fed on TuYV infected *C. b-p* plants for 18–24 h, after which they were transferred to 2–3 week old healthy *Nicotiana benthamiana* seedlings for a 4 day inoculation access period (IAP), after which aphids were killed by spraying with BioAdvanced 3-In-1 Insect, Disease, and Mite Control (Bayer). The plants were kept in an air conditioned room until they grew large enough for agro-inoculation (4–6 leaf stage).

**Table 1:** Single and mixed virus infections investigated in this study.

Single	Double	Triple
TuYV	TuYV + CMoV	TuYV + CMoV + Gamma
CMoV	TuYV + Gamma	TuYV + CMoV + Sigma
Gamma	TuYV + Sigma	TuYV + CMoV + ST9
Sigma	TuYV + ST9	
ST9	CMoV + Gamma	
	CMoV + Sigma	
	CMoV + ST9	

TuYV is the polerovirus, turnip yellows virus, CMoV is the umbravirus, carrot mottle virus, and Gamma, Sigma, and ST9 refer to the specific tombusvirus-like associated RNAs (tlaRNAs).

For CMoV and tlaRNA inoculations, cultures of *A. tumefaciens* strain GV3101, transformed individually with infectious clones of each virus, were prepared as described by Erickson et al. (2023); cultures resuspended in infiltration buffer were adjusted to an optical density at 600 nm (O.D._600_) of 1.0. The *A. tumefaciens* cultures were infiltrated using a needless syringe into 3–4 leaves of healthy or TuYV-inoculated *N. benthamiana* plants at the 4–6 leaf stage; for treatments including both CMoV and a tlaRNA, cultures were mixed 1:1 prior to infiltration. The plants were maintained in the air conditioned room for 4–5 days post inoculation (dpi) then transferred to a growth chamber kept at 19°C, 70% relative humidity (RH), and 16:8 h light:dark photoperiod. After 3 weeks post infection (wpi) tissue was collected and stored at −80°C prior to RNA extraction and RT-qPCR analysis. For treatments without tlaRNAs, tissue was only collected from non-inoculated leaves; for treatments with a tlaRNA, tissue was collected from both inoculated and systemic leaves. The experiments were repeated twice.

### Aphid transmission experiments

To determine how different infection combinations affected the ability of each of the viruses in this study to be aphid transmitted, aphid transmission experiments were conducted as described in the previous section, this time using as the inoculum source leaf tissue from healthy plants and plants infected with the various single and multi-virus combinations described above. For treatments with a tlaRNA alone, TuYV with any tlaRNA, or CMoV + Gamma, agroinoculated leaves were used as the inoculum source; for all other treatments non-inoculated leaf tissue was used. Inoculated plants were maintained under the same conditions as the plants described in the previous section. After 4 wpi symptoms were noted and leaf tissue was collected and stored at −80°C prior to RNA extraction and RT-qPCR analysis. The experiments were repeated twice.

### Mechanical transmission experiments

To determine how different infection combinations affected the ability of each of the viruses in this study to be mechanically transmitted, leaf tissue from healthy plants and plants harboring each of the described virus infection treatments was ground with a mortar and pestle in 0.03 M KPO_4_ + 0.1% Na_2_SO_3_ (pH 7) buffer in a 1:6 ratio (w/v), with celite added as an abrasive. For treatments with tlaRNAs alone, TuYV + tlaRNAs, or CMoV + Gamma, infiltrated leaves were used as the inoculum source, for all other treatments non-inoculated leaves were used. Using a sterile cotton swab, the homogenized plant sap was gently rubbed onto 3–4 leaves of healthy *N. benthamiana* plants at the 4–6 leaf stage. Plants were kept in a growth chamber held at 19°C, 70% relative humidity (RH), and 16:8 h light:dark photoperiod. At about 3–4 wpi, symptoms were noted and leaf tissue was collected and stored at −80°C prior to RNA extraction and RT-qPCR analysis. The experiments were repeated twice.

### Multiplexed RT-qPCR assay validation

Primer and probe sequences specific to each virus used in this study were designed using the PrimerQuest Tool.^1^ Standard curves were made using plasmids harboring cloned viral genome sequences of each virus to determine the amplification efficiency of each primer/probe set (without fluorophore); temperature gradient analysis was also performed to determine an optimal annealing temperature. The assays were performed on the CFX96 Touch Real-Time PCR Detection System (Bio-Rad), in a reaction mix containing 10 μL of SsoAdvanced Universal SYBR® Green Supermix (Bio-Rad) 0.6 μL of each primer (10 μM), 2 μL of template, and 6.8 μL of nuclease free water. The thermocycling conditions were 95°C for 3 m followed by 40 cycles of 95°C for 10 s and 55°C–65°C for 30 s, and concluded with melt curve analysis; a shared optimal temperature of 60°C was selected. Each primer set was tested against non-target virus plasmids to confirm primer specificity. Next, plasmids with each virus were pooled in equimolar amounts, and standard curve analysis was repeated to confirm the pooled sample had minimal effects on primer amplification efficiency.

Primer and probes (with fluorophore) were validated—first individually, then in a multiplexed reaction—in the same manner (excluding temperature gradient and melt-curve analyses), using a reaction mix containing 10 μL of iQ Multiplex Powermix (Bio-Rad), 0.04 μL of each primer (100 μM), 0.02 μL of probe (100 μM), 2 μL of template, and nuclease free water to 20 μL, and the same thermocycling conditions. The amplification efficiency of all sets was between 90% and 110%. All primers and probes used in this study are listed in Supplementary Table S1.

### RNA extraction and multiplexed RT-qPCR analysis

Total RNA was extracted from leaf tissue samples using TRIzol™ Reagent (Invitrogen) according to the manufacturer’s protocol. For the initial experiments in which the different single and multi-virus infections were established, samples were treated with RQ1 RNase-Free DNase (Promega) and cleaned by phenol/chloroform extraction prior to cDNA synthesis; this was not done for samples from the aphid and mechanical transmission experiments. RNA was used as template with the iScript™ cDNA Synthesis Kit (Bio-Rad); the synthesized cDNA was diluted to 5 ng/μL with nuclease free water. The qPCR reaction contained 10 μL of iQ Multiplex Powermix, 0.04 μL of each primer (100 μM), 0.02 μL of each prober (100 μM), 2 μL (10 ng) of cDNA template, and nuclease free water to 20 μL, and the thermocycling conditions were as described in the previous section. The cytochrome C oxidase gene was used as a reference. Two technical replicates were performed for each sample. The 2^–∆∆^ Ct method was used to calculate the relative viral accumulation in the initial set of experiments establishing the different virus infection treatments.

### Statistical analyses

Significant differences in viral accumulation between different single and mixed virus infections were determined using ANOVA using generalized linear models with the corresponding R packages in InfoStat v2008. Normality and homoscedasticity were checked and corrected when necessary and means were separated using Fisher’s least significant difference test (*p <* 0.05). Data was plotted in GraphPad Prism v.5.03.

## Results

### Symptom development

**Figure 2:**
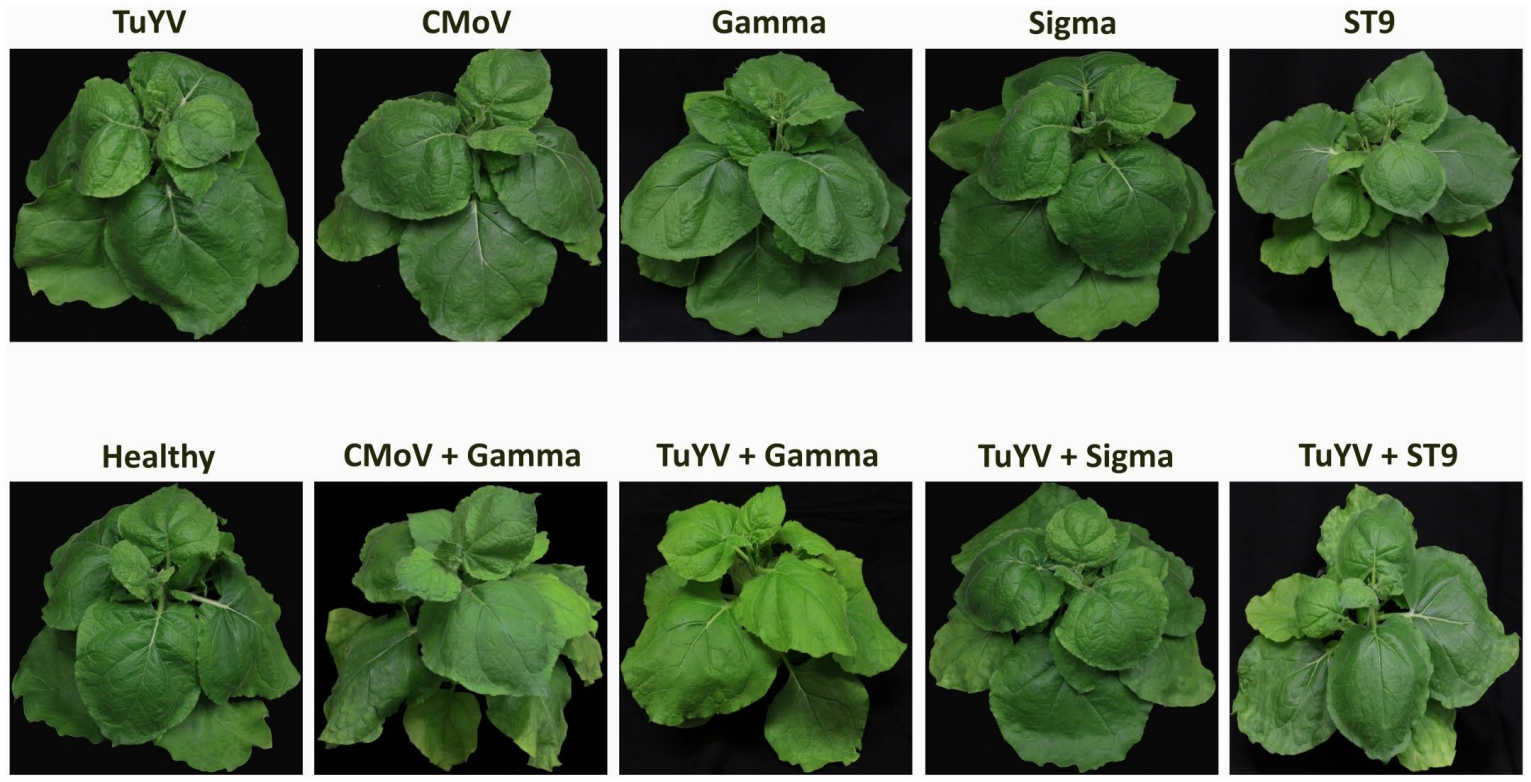
Asymptomatic virus infections in *Nicotiana benthamiana* plants. Depicted are asymptomatic *N. benthamiana* plants that have been inoculated singly with TuYV, CMoV, and tlaRNAs Gamma, Sigma, or ST9, doubly with TuYV and each of the tlaRNAs, and inoculated with CMoV + Gamma, along with a healthy, non-inoculated plant for comparison. Labels above each plant picture indicate the virus infection treatment. Photos were taken 3 wpi.

**Figure 3:**
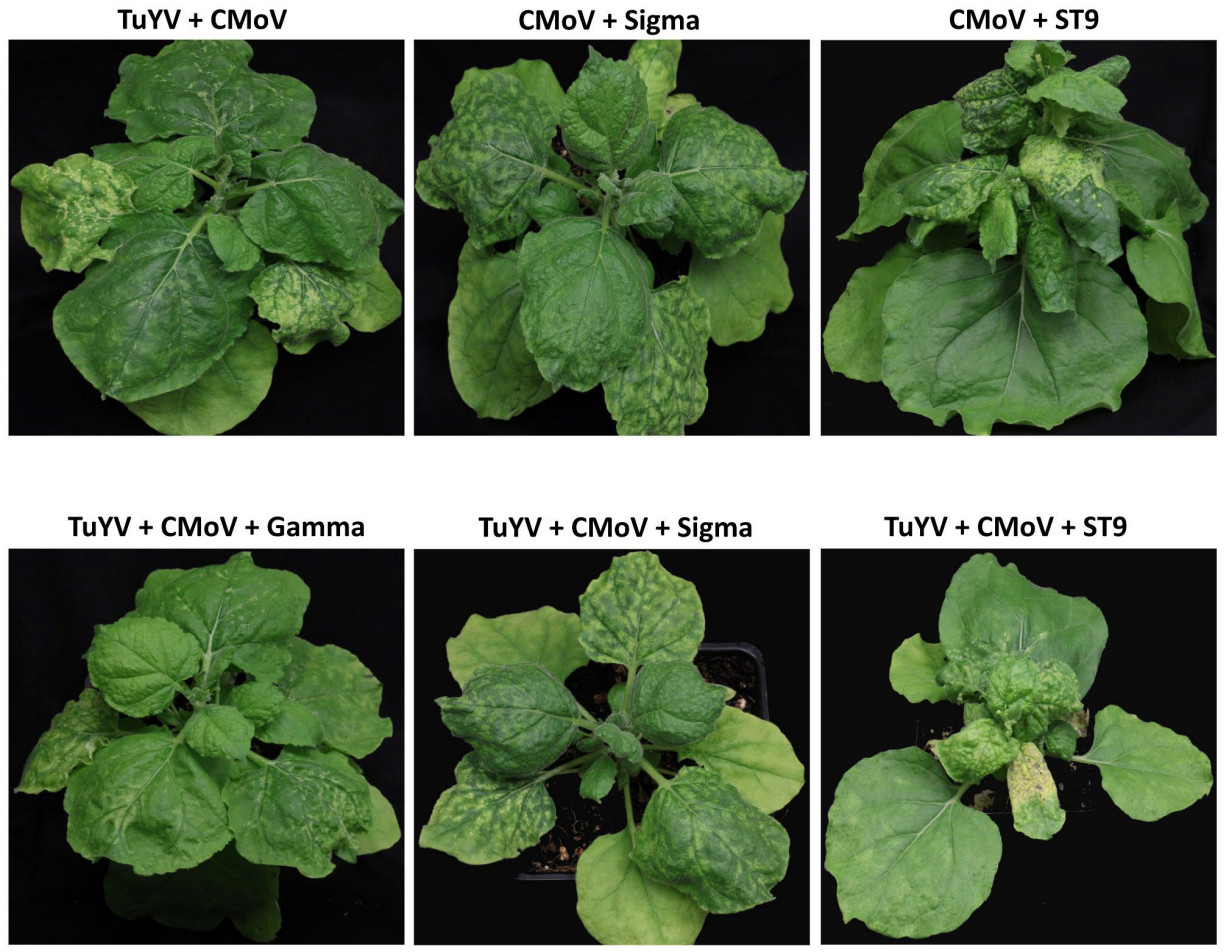
Symptomatic virus infections in *Nicotiana benthamiana* plants. Depicted are asymptomatic plants doubly infected with CMoV and either Sigma or ST9, or CMoV and TuYV, and plants triply infected with TuYV, CMoV, and either Sigma or ST9. Labels above each plant picture indicate the virus infection treatment. Photos were taken 3 wpi.

**Figure 4:**
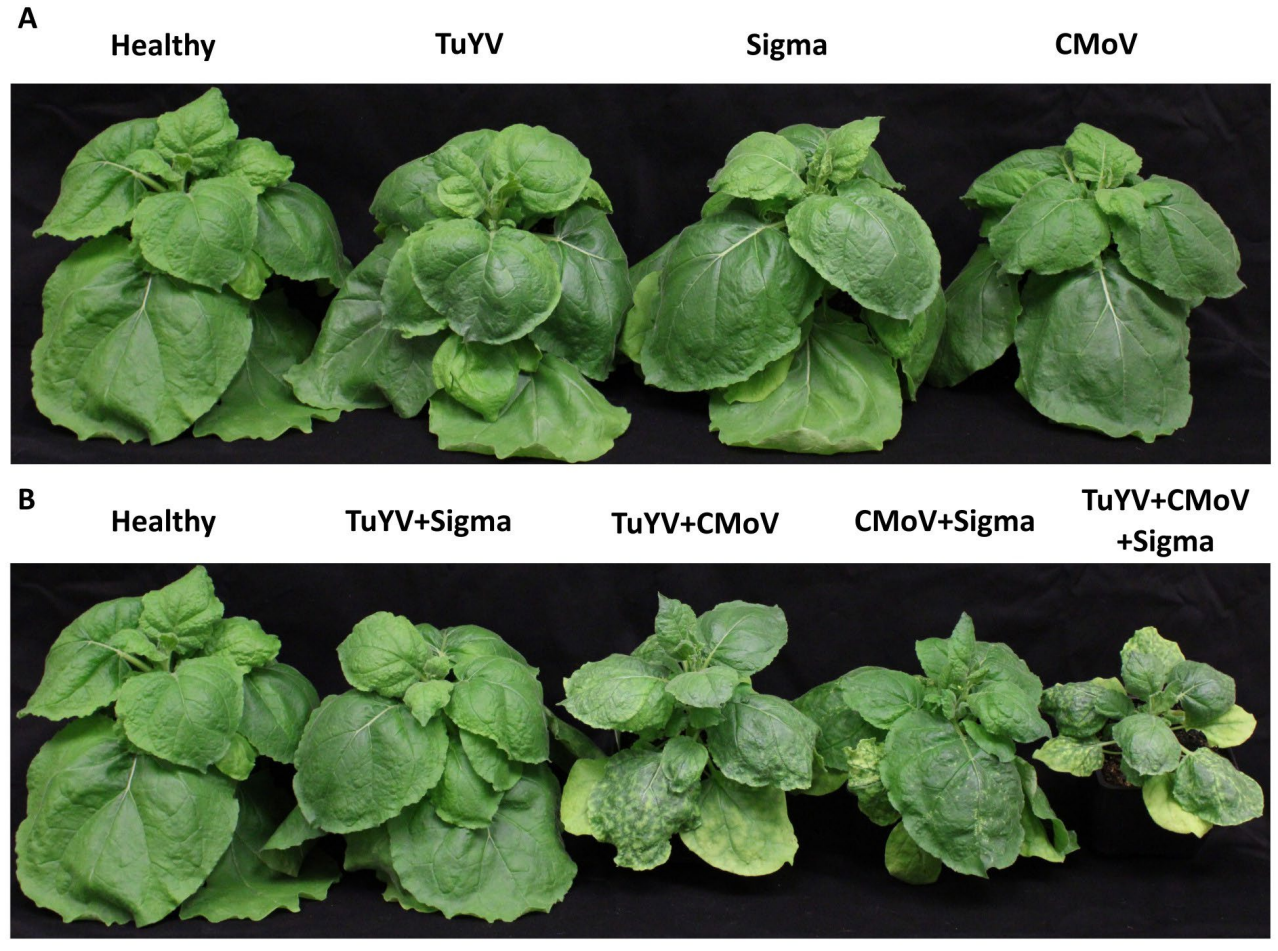
Lineup of virus inoculated *Nicotiana benthamiana* plants. Depicted are *Nicotiana benthamiana* plants harboring single or multi-virus infections of TuYV, CMoV, and/or Sigma. **(A)** Asymptomatic virus infections; little to no difference in plant stature was observed between the different virus treatments represented. **(B)** Symptomatic virus infections; notable stunting can be seen in plants infected with 2 or more viruses, relative to a healthy control. A similar, albeit more severe, effect was observed for plants harboring co-infections including ST9, whereas this effect was not observed in plants harboring co-infections including Gamma. Labels above each plant picture indicate the virus infection treatment. Photos were taken 3 wpi.

No notable symptoms were observed in plants singly infected with any of the viruses, in any plants doubly infected with TuYV and any of the tlaRNAs, or in plants infected with CMoV + Gamma (Figure 2). Plants co-infected with CMoV + Sigma or with CMoV + ST9 developed prominent leaf mosaic symptoms, which were more severe in CMoV + ST9 infected plants which displayed more prominent yellowing as well as mild leaf curling. Plants infected with TuYV + CMoV developed punctate necrotic spots on the leaves, along with mild mosaic symptoms. These same symptoms were observed in plants co-inoculated with TuYV + CMoV + Gamma. In plants co-inoculated with TuYV + CMoV + Sigma or + ST9, the observed symptoms appeared to be a combination of those that were observed in the CMoV + TuYV, CMoV + Sigma, and CMoV + ST9 plants, displaying dramatic leaf mosaic, chlorosis, and punctate necrotic lesion symptoms on leaves (Figure 3). For all non-symptomatic plants and plants co-inoculated with CMoV + Sigma or + ST9, no prominent differences in overall growth were observed (Figure 4A). However, plants co-inoculated with TuYV + CMoV, and TuYV + CMoV + Sigma or + ST9 were severely stunted, and this effect was most severe in the TuYV + CMoV + ST9 co-infected plants (Figure 4B).

### Systemic trafficking of tlaRNAs

In plants singly infected with each tlaRNA, none of the tlaRNAs could be detected in the upper non-inoculated leaves, despite being detected in the inoculated leaves. This was also true for plants harboring co-infections of TuYV + Gamma or TuYV + Sigma. In a few plants, while TuYV was detected in non-inoculated leaves, it was not detected in leaves agroinoculated with the tlaRNA, which may explain the lack of tlaRNA systemic movement in these plants. However, in the majority of the plants tested both TuYV and the tlaRNAs were detected in inoculated leaves, while only TuYV was detected in the upper non-inoculated indicating that, under the conditions used in this study, TuYV did not support systemic trafficking of these tlaRNAs. However, in plants co-infected with TuYV + ST9 both viruses were detected in the upper non-inoculated leaves of three plants (and in the inoculated leaves of all eight plants tested).

In plants infected with CMoV in combination with each of the tlaRNAs, all of the tlaRNAs could be detected in the upper non-inoculated leaves, although this interaction occurred with differing frequencies for the different tlaRNAs. In CMoV + Sigma and CMoV + ST9 co-infected plants, both tlaRNAs were systemically trafficked 100% of the time, however, in CMoV + Gamma co-infected plants, systemic trafficking of Gamma was only observed 50% of the time. Similar results were observed for plants co-infected with TuYV, CMoV, and each of the tlaRNAs. These results are summarized in Table 2.

**Table 2:** Summary of symptom development and tlaRNA systemic movement in the various single and mixed virus infections established in *Nicotiana benthamiana.*

		Single infection					Double infection							Triple infection		
Infecting viruses		TuYV	CMoV	Gamma	Sigma	ST9	TuYV + Gamma	TuYV + Sigma	TuYV + ST9	TuYV + CMoV	CMoV + Gamma	CMoV + Sigma	CMoV + Gamma	TuYV + CMoV + Gamma	TuYV + CMoV + Sigma	TuYV + CMoV + ST9
Symptoms		No	No	No	No	No	No	No	No	Yes	No	Yes	Yes	Yes	Yes	Yes
tlaRNA systemic movement		-	-	0/10	0/8	0/8	0/10	0/7	3/8	-	4/10	8/8	8/8	4/10	8/8	8/8
No. of plants infected	TuYV	22/23	-	-	-	-	10/10	7/7	8/8	22/23	-	-	-	10/10	8/8	8/8
	CMoV	-	21/23	-	-	-	-	-	-	23/23	9/10	8/8	8/8	10/10	8/8	8/8
	tlaRNA	-	-	10/10	8/8	8/8	10/10	7/7	8/8	-	10/10	8/8	8/8	10/10	8/8	8/8

The top row of the table indicates the infecting virus(es), the second row indicates whether symptoms were observed in plants infected with the respective single and co-infection treatments, and the third row indicates the number of plants in which the co-infecting tlaRNA could be detected in the upper non-inoculated leaves. The bottom three rows of the table indicate the infection rates (number of plants infected/number of plants inoculated) for all of the viruses in each treatment.

### Relative virus accumulation

**Figure 5:**
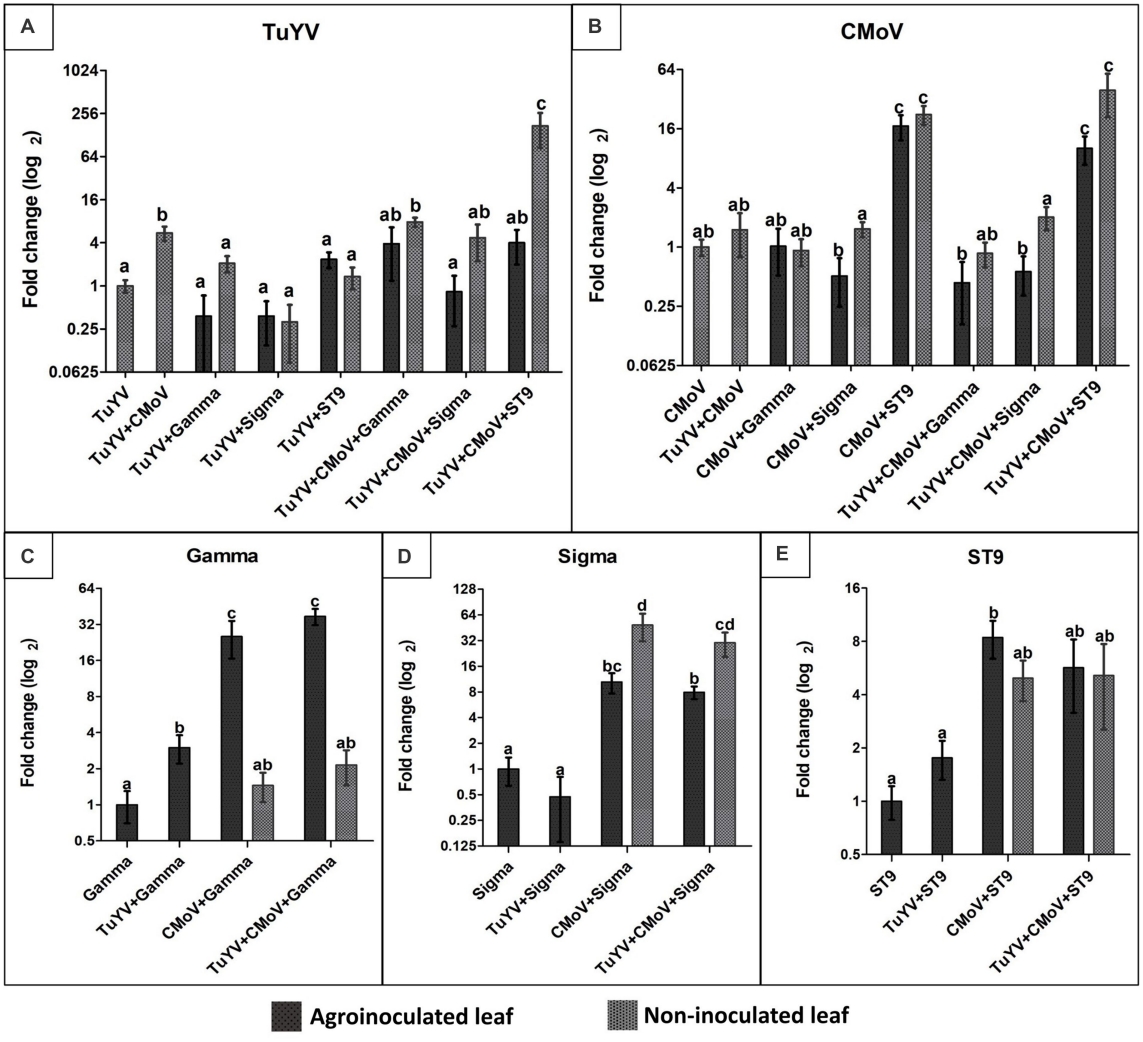
Relative accumulation of TuYV, CMoV, and tlaRNAs Gamma, Sigma, and ST9 as a function of co-infection. Graphs depict log_2_ changes in viral accumulation of **(A)** TuYV, **(B)** CMoV, and the tlaRNAs **(C)** Gamma, **(D)** Sigma, and **(E)** ST9 in mixed infections relative to accumulation of each of these viruses in single infections; the y-axis. Virus accumulation was quantified using RT-qPCR and calculated using the 2^–∆∆^ Ct method. Black and gray bars represent relative viral accumulation in agroinoculated and non-inoculated leaves, respectively. Graphs depict the means ± SEs. Significant differences between treatments were determined using ANOVA with a significance value of *p* < 0.05; different letters indicate significant differences between treatments, whereas shared letters indicate there was not a significant difference between treatments.

The only co-infection treatments which were associated with a significant increase in TuYV accumulation relative to plants infected with TuYV alone were co-infections of TuYV + CMoV (5.50-fold increase), TuYV + CMoV + Gamma [7.84-fold increase in non-inoculated leaves (NILs)], and by far the most dramatic increase (172.66-fold) was observed in the NILs of TuYV + CMoV + ST9 infected plants. All other co-infection treatments resulted in non-significant changes in TuYV accumulation (Figure 5A). CMoV accumulation increased significantly in the inoculated leaves (ILs; 17.08- and 10.10-fold) and NILs (22.36- and 39.48-fold) of CMoV + ST9 and TUYV + CMoV + ST9 inoculated plants, respectively. All other infection treatments produced minimal, non-significant changes in CMoV accumulation with respect to accumulation levels in plants inoculated with only CMoV (Figure 5B).

Gamma accumulation levels increased significantly in the ILs of TuYV + Gamma, CMoV + Gamma, and TuYV + CMoV + Gamma inoculated plants by 3.00-, 25.35-, and 37.25-fold, respectively. Interestingly in the NILs of CMoV + Gamma and TuYV + CMoV + Gamma, Gamma accumulation levels (1.45- and 2.15-fold, respectively) did not vary significantly from that in the ILs of plants inoculated with Gamma alone (Figure 5C). Among the tlaRNAs tested in this study, Sigma accumulation varied the most in co-infected plants, relative to that in the ILs of plants inoculated with Sigma alone. While Sigma accumulation did not change significantly in the ILs of TuYV + Sigma infected plants (0.55-fold), it increased significantly both in the ILs (10.47- and 7.9-fold) and the NILs (48.66- and 30.16-fold) of CMoV + Sigma and TuYV + CMoV + Sigma co-infected plants, respectively (Figure 5D). ST9 accumulation levels were not significantly altered in the ILs of TuYV + ST9 infected plants, and only varied significantly in the ILs of CMoV + ST9 inoculated plants (1.76-fold increase), however a noticeable but non-significant increasing trend was observed in the NILs of these plants (4.96-fold), as well as in the ILs (5.68-fold) and NILs (5.13-fold) of TuYV + CMoV + ST9 inoculated plants (Figure 5E). It is interesting to note that in some co-infections with ST9, TuYV and CMoV accumulations both dramatically increased, but a compensatory increase in ST9 accumulation was not observed.

### Effects of co-infection on mechanical transmission

As expected, when *N. benthamiana* plants were inoculated using tissue from plants infected with any of the viruses in this study not known to be independently mechanically transmissible (TuYV and tlaRNAs), systemic infections were not observed by any of these viruses; when the rub-inoculated leaves were tested for these viruses, low level amplification of all but Gamma could be detected. This low level detection may simply be attributed to residual inoculum, or it may suggest that some cells could become infected with these viruses by rub inoculation but that the viruses could not move beyond the inoculated cells (data not shown); these results coincide with those from early studies on ST9 in rub inoculated *C. b-p* leaves (Passmore et al., 1993). CMoV was mechanically transmitted and initiated a systemic infection in 100% of the rub-inoculated plants, regardless of whether the inoculum source was infected with CMoV alone or in combination with any of the other viruses. Somewhat unexpectedly, Sigma and ST9 were both efficiently mechanically transmitted (and established systemic infections) from plants co-infected with either of these tlaRNAs and CMoV, and plants co-infected with TuYV + CMoV and either of these tlaRNAs; Sigma was transmitted to 100 and 69%, respectively, of plants when tissue from CMoV + Sigma and TuYV + CMoV + Sigma co-infected plants were used as the inoculum source, and ST9 was transmitted to 100 and 62%, respectively, of plants when tissue from CMoV + ST9 and TuYV + CMoV + ST9 co-inoculated plants, were used as the inoculum source. Gamma could not be detected in the ILs or NILS of any rub inoculated plants, regardless of whether tissue from CMoV + Gamma or TuYV + CMoV + Gamma infected plants was used as the inoculum source. TuYV was transmitted with very low efficiency (11%) from TuYV + CMoV co-infected plants. Interestingly, the efficiency with which TuYV was mechanically transmitted increased markedly from plants co-infected with TuYV + CMoV and either Sigma or ST9. When TuYV + CMoV + Sigma infected plants were used as the inoculum, the transmission rate of TuYV increased to 54%, and when TuYV + CMoV + ST9 infected plants were used as the inoculum source it increased to 77%. This data is summarized in Table 3.

**Table 3:** Summary of the mechanical transmission rates of TuYV, CMoV, and tlaRNAs from *Nicotiana benthamiana* plants infected with different combinations of each of these viruses.

		Single-virus infection					Double-virus infection							Triple-virus infection		
Viruses in inoculum		TuYV	CMoV	Gamma	Sigma	ST9	TuYV + Gamma	TuYV + Sigma	TuYV + ST9	TuYV + CMoV	CMoV + Gamma	CMoV + Sigma	CMoV + ST9	TuYV + CMoV + Gamma	TuYV + CMoV + Sigma	TuYV + CMoV + ST9
No. of plants infected (%)	TuYV	0/18	-	-	-	-	0/23	0/23	0/23	2/18	-	-	-	0/15	7/13	10/13
		0%					0%	0%	0%	11%				0%	54%	77%
	CMoV	-	-	-	-	18/18	-	-	-	18/18	15/15	13/13	13/13	15/15	13/13	13/13
						100%				100%	100%	100%	100%	100%	100%	100%
	tlaRNA	-	0/23	0/23	0/23	-	0/23	0/23	0/23	-	0/15	13/13	13/13	0/15	9/13	8/13
			0%	0%	0%		0%	0%	0%		0%	100%	100%	0%	69%	62%

The top row of the table indicates which viruses were present in the inoculum tissue used for rub inoculation, and the succeeding rows indicate the transmission rate of each virus in each treatment in terms of number of plants infected/number of plants inoculated (white row) and in percentage of plants infected (gray row).

### Effects of co-infection on aphid transmission

As expected, none of the viruses in this study not known to be independently aphid transmitted (CMoV and tlaRNAs) could be detected in aphid inoculated plants, when tissue from plants infected singly or in combination with any of these viruses was used as the inoculum. Co-infection with TuYV did facilitate aphid transmission of CMoV when plants co-infected with TuYV + CMoV, TuYV + CMoV + Gamma, and TuYV + CMoV + ST9 were used as the inoculum source—CMoV was transmitted to 42%, 43%, and 64% of recipient plants, respectively, and TuYV was, respectively, transmitted to 100%, 77%, and 77% of recipient plants. Neither Gamma nor Sigma became aphid transmissible when co-infected with TuYV, despite TuYV being transmitted to 100% and 92% of recipient plants from these inoculum sources. Triple infections of each of these tlaRNAs with TuYV and CMoV did not yield different results, despite TuYV transmission efficiency remaining high (100% and 77%, respectively). Conversely ST9 was successfully aphid transmitted with low efficiency (10%) to a single recipient plant from plants co-infected with TuYV + ST9, and was aphid transmitted to 7% of recipient plants when the inoculum source came from plants also infected with TuYV + CMoV. Transmission efficiencies of TuYV in these treatments were 100% and 77%, respectively. These results are summarized in Table 4.

**Table 4:** Summary of the aphid transmission rates of TuYV, CMoV, and tlaRNAs from *Nicotiana benthamiana* plants infected with different combinations of each of these viruses.

		Single-virus infection					Double-virus infection							Triple-virus infection		
Viruses in inoculum		TuYV	CMoV	Gamma	Sigma	ST9	TuYV + Gamma	TuYV + Sigma	TuYV + ST9	TuYV + CMoV	CMoV + Gamma	CMoV + Sigma	CMoV + ST9	TuYV + CMoV + Gamma	TuYV+ CMoV + Sigma	TuYV + CMoV + ST9
No. of plants infected (%)	TuYV	20/25	-	-	-	-	13/13	11/12	10/10	22/24	-	-	-	14/14	10/13	13/14
		80%					100%	92%	100%	92%				100%	77%	77%
	CMoV	-	-	0/25	-	-	-	-	-	10/24	0/15	0/15	0/14	6/14	0/13	9/14
				0%						42%	0%	0%	0%	43%	0%	64%
	tlaRNA	-	0/15		0/14	0/15	0/13	0/12	1/10	-	0/15	0/15	0/14	0/14	0/13	1/14
			0%		0%	0%	0%	0%	1%		0%	0%	0%	0%	0%	7%

The top row of the table indicates which viruses were present in the inoculum tissue used for aphid inoculation, and the succeeding rows indicate the transmission rate of each virus in each treatment in terms of number of plants infected/number of plants inoculated (white row) and in percentage of plants infected (gray row).

## Discussion

While there are many studies on polerovirus-tlaRNA and polerovirus-umbravirus interactions in disease complexes harboring various combinations of these viruses, there exist only two recent studies that have begun to touch upon umbravirus-tlaRNA interactions (Yoshida, 2020; Chen et al., 2022). While we investigated the effects of co-infection in all possible co-infection combinations of the polerovirus TuYV, the umbravirus CMoV, and the tlaRNAs Gamma, Sigma, and ST9—with respect to single infections of each of these viruses—perhaps the most intriguing findings we uncovered were those concerning interactions involving CMoV and tlaRNAs.

With respect to symptom development in *N. benthamiana* plants, the most interesting result we found was that almost all co-infections that included CMoV (with the exception of CMoV + Gamma) induced enhanced symptom development in inoculated plants relative to those infected with any virus alone or plants co-infected with TuYV and any tlaRNA. Additionally, there were noticeable differences in symptom presentation depending on if CMoV was co-infected with TuYV (dispersed, punctate, necrotic lesions on leaves) or with either Sigma or ST9 (mosaic symptoms on leaves). There were also notable differences in the severity of leaf mosaic symptoms between co-infections that included Sigma (less severe) and those that included ST9 (more severe), both in double infections with CMoV and in triple infections with TuYV and CMoV. These results suggest that, in this model host and disease complex system, CMoV is the key driver of symptom development since symptoms only occurred in co-infections in which it was present. These results also demonstrate marked differences in symptom development with respect to each of the tlaRNAs; as more of these tlaRNAs are being regularly discovered, it will be interesting to further uncover the various ways they differ in the effects they have on symptom development and interactions they have with co-infecting viruses in these unique disease complexes.

Until recently, it was thought that only poleroviruses were responsible for systemically trafficking tlaRNAs in these disease complexes. However, a recent study by Yoshida (2020) found that after attempting to aphid transmit CRLV, CMoV, and a CRLVaRNA from CMD affected carrot plants harboring all three of these viruses to Japanese parsley (*Cryptotaenia canadensis* subsp. *Japonica*), only CMoV and the CRLVaRNA could be detected in the recipient plant, making this the first reported evidence that an umbravirus may support tlaRNA systemic movement in the absence of a co-infecting polerovirus, although these results could also have other potential explanations. In another recent study by Chen et al. (2022), after co-agroinoculation of infectious clones of the umbravirus tobacco bushy top virus (TBTV) with the tobacco vein distorting virus associated RNA (TVDVaRNA) the authors could detect the TVDVaRNA along with TBTV in the distal non-inoculated leaves, thereby confirming an umbravirus could independently support tlaRNA systemic movement. Here, we present data demonstrating that CMoV was able to support systemic movement of all three tlaRNAs (Gamma, Sigma, ST9), however this interaction appeared to be less efficient in co-infections with Gamma, suggesting some degree of specificity in these umbravirus-tlaRNA interactions.

Since neither Gamma nor Sigma moved systemically when co-infected with TuYV alone, it is possible there exists a degree of specificity in polerovirus-tlaRNA interactions. However, in TuYV + ST9 co-infected *N. benthamiana* plants, ST9 only moved systemically 38% of the time, which was odd as these viruses are known to naturally co-occur and form a strong helper-dependence relationship in *Capsella bursa-pastoris* plants (Falk and Duffus, 1984; Passmore et al., 1993; Sanger et al., 1994). We speculated this discrepancy might result from combined aphid inoculation of TuYV and agroinoculation of ST9 effectively failing to introduce these two viruses into the same cells. To address this we conducted preliminary experiments in which we coinfiltrated each of the tlaRNAs with an infectious clone of another polerovirus [barley virus G (BVG)] that we had on hand (Erickson et al., 2023). Interestingly, ST9 could be detected along with BVG in the upper NILs in 100% of co-infected plants; neither Gamma nor Sigma were detected in the upper non-inoculated leaves, suggesting that potential specificity in polerovirus + tlaRNA interactions may be driven by the tlaRNA (Supplementary Figure S1).

In several of these virus disease complexes, it has been found that co-infection increases viral RNA accumulation of one or more of the co-infecting viruses (Sanger et al., 1994; Yoshida, 2020; Chen et al., 2022). Our results shows that tlaRNA ST9 appears to have a significant impact on CMoV accumulation, both in CMoV + ST9 and TuYV + CMoV + ST9 co-infections, and on TuYV accumulation in TUYV + CMoV + ST9 co-infected plants. These dramatic increases in CMoV and TuYV accumulation could potentially explain why symptom development was most severe in plants harboring these co-infection combinations. Surprisingly, TuYV + ST9 co-infection in *N. benthamiana* plants did not stimulate a significant increase in TuYV accumulation, which was again unexpected given that in natural TuYV + ST9 co-infections in *C. bursa-pastoris*, the accumulation of both TuYV genomic RNAs and capsid proteins significantly increased (Falk and Duffus, 1984; Passmore et al., 1993; Sanger et al., 1994). This discrepancy may again indicate a requirement for certain host factor(s) to facilitate TuYV + ST9 interactions.

Interestingly, the relative accumulation of Gamma increased significantly in the ILs of CMoV + Gamma and TuYV + CMoV + Gamma co-infected plants, but minimal differences in Gamma accumulation were observed in the NILs. A somewhat similar effect was observed for ST9 in CMoV + ST9 infected plants, wherein a significant increase was observed in ILs but not in NILs of CMoV + ST9 inoculated plants, however there was a notable increasing trend of ST9 in the NILs. The opposite was observed for Sigma, wherein a non-significant increasing trend in Sigma accumulation was observed in the ILs of CMoV + TuYV and TuYV + CMoV + ST9 inoculated plants, while a significant increase was observed in the NILs; this overall increase in accumulation of Sigma may partially explain the enhanced mosaic leaf symptoms observed in these co-infected plants.

The mechanisms responsible for increased accumulation of some viruses as a result of co-infection aren’t precisely known, however there are three main ways this is thought—and in some instances has been demonstrated—to occur (Rochow, 1972; Latham and Wilson, 2008; Syller, 2012; Alcaide et al., 2020). One is that co-infection functions to increase the replication of one or more co-infecting viruses, resulting in more viral copies per cell, as has been found in co-infections of the plant infecting reoviruses southern rice-black streaked dwarf virus (SRBSDV) and rice ragged stunt virus (RRSV), and for TuYV and ST9 in *C. b-p.* plants (Passmore et al., 1993; Li et al., 2017). Another possibility is that umbravirus encoded movement proteins interact with co-infecting heterologous viral RNAs to impart them with cell-to-cell and systemic movement within the plant thereby resulting in more cells being infected with the dependent virus, as has been observed in co-infections of the polerovirus potato leafroll virus (PLRV) with the umbravirus pea enation mosaic virus 2 (PEMV2; Ryabov et al., 2001a). This could explain the increase of TuYV accumulation in the presence of CMoV, since on its own TuYV is phloem limited and co-infection may help it break this phloem limitation. Weak interactions between Gamma RNAs and CMoV movement proteins may explain the differences in accumulation of Gamma between the ILs and NILs of plants co-infected with CMoV, as well as the reduced efficiency of systemic transport of Gamma. A third possibility is that one or more of the co-infecting viruses have different host defense mechanisms that can suppress host defense systems against which the other co-infecting virus(es) may be susceptible. For example, the P0 protein of some poleroviruses, including TuYV, functions as a suppressor of the RNA interference (RNAi) system of the host plant (Baumberger et al., 2007; Bortolamiol et al., 2007; Csorba et al., 2010). TlaRNA ST9 was found to have a structural feature in the 3′ untranslated region (UTR) of its genomic RNA that functions to stall host XRN1 degradation (Campbell et al., 2022), which may explain the dramatic effect ST9 appeared to have on TuYV and CMoV accumulation in co-infected plants. The long distance movement protein (encoded by ORF3) of umbraviruses has been shown to form protective ribonucleoprotein complexes with both umbravirus and heterologous virus RNAs (Ryabov et al., 2001b; Taliansky et al., 2003), and has also been shown in PEMV2 to protect viral RNAs from the host nonsense mediated decay (NMD) degradation pathway (May et al., 2020). Perhaps the combined effects of the TuYV P0 RNAi silencing suppressor (RSS) activity, putative CMoV derived NMD resistance, and stalling of XRN1 degradation by ST9 could explain the drastic increases of TuYV and CMoV in co-infections of these three viruses and the severe symptom development. It is likely that a combination of these various viral functions interplay to produce the variable effects on viral accumulation and disease presentation observed in the different co-infections of the viruses used in this study.

There are multiple examples of umbraviruses conferring mechanical transmissibility to a co-infecting polerovirus. Falk et al. (1979) showed that TuYV [formerly referred to as beet western yellows virus (BWYV)] was occasionally mechanically transmitted from plants co-infected with the umbravirus, lettuce speckles mottle virus (LSMV), and PLRV has been observed to gain mechanical transmissibility as a result of co-infection with PEMV2 (Falk et al., 1979; Ryabov et al., 2001a). In the latter study, it was also found that PLRV became mechanically transmissible when co-infected with a cucumber mosaic virus (CMV) vector engineered to express the GRV ORF4 protein, suggesting that this protein likely plays an important mechanistic role in mechanical transmission. However, when PLRV was coinoculated with a GRV-ORF4 expressing CMV vector that had a defective 2b RSS, mechanical transmissibility of PLRV was lost, further highlighting the likely role virus encoded host defense suppressors may play in such interactions. However, there are other examples of polerovirus-umbravirus co-infections that did not confer mechanical transmissibility to the polerovirus, as has been observed in co-infections of tobacco bushy top virus (TBTV) and tobacco vein distorting virus (TVDV; Chen et al., 2022). While it has been speculated, no studies have been published on whether an umbravirus can confer mechanical transmissibility to a tlaRNA, until now. In this study, we found that both Sigma and ST9 could be mechanically transmitted from plants co-inoculated with CMoV; Gamma, conversely, did not gain mechanical transmissibility.

When TuYV was co-infected with CMoV alone, it became mechanically transmissible, but with extremely low efficiency (11%). However, when TuYV was co-inoculated with CMoV and either Sigma or ST9, the efficiency of TuYV mechanical transmission greatly increased. The observed increase in TuYV accumulation in these plants may partially explain the increased rate of mechanical transmission of TuYV. Conversely, the low accumulation of Gamma may help to explain why this tlaRNA could not be mechanically co-transmitted with CMoV.

We also found that CMoV became aphid transmissible when co-infected with TuYV, except from plants co-infected with TuYV + CMoV + Sigma. This supports similar findings that demonstrate CMoV could be transmitted by *M. persicae* aphids when co-infected with either of the poleroviruses potato leaf roll virus (PLRV) or beet western yellows virus (BWYV), along with other examples of compatible interactions between non-naturally co-occurring poleroviruses and umbraviruses, which could have important epidemiological implications for the development of novel disease complexes or transmission of umbraviruses to novel hosts (Abraham et al., 2014; Zhou et al., 2017). CMoV was transmitted with the greatest efficiency (64%) from plants co-infected with TuYV + CMoV + ST9. Whether this increase in CMoV co-transmission rate in the presence of ST9, or the lack of CMoV co-transmission observed when Sigma was present, are indicative of potential synergistic and antagonistic effects, respectively, requires further investigation. Similar to other findings in this study, while ST9 did become aphid transmissible when co-infected with TuYV, the transmission rate was lower than expected, again highlighting the potential need of host specific factors for this interaction.

Together, these findings add to the growing body of knowledge on disease complexes involving these types of viruses and provide novel insights into the virus-virus interactions that occur. Such information could potentially be employed in programs aimed at preventing or controlling outbreaks of such disease complexes and perhaps could be used in the development of novel virus-based gene delivery systems.

## Data availability statement

The original contributions presented in the study are included in the article/Supplementary material, further inquiries can be directed to the corresponding author.

## Author contributions

AE designed and executed the experiments, analyzed the data, and wrote the manuscript. BF reviewed and revised the manuscript. All authors contributed to the article and approved the submitted version.

## Footnotes

^1^ www.idtdna.com/PrimerQuest/Home/Index

## Supplementary material

SUPPLEMENTARY FIGURE S1 RT-PCR data of from BVG + tlaRNA co-inoculation experiments. Panel **(A)** shows the products obtained from RT-PCR based detection of BVG in *Nicotiana benthamiana* plants that were agroinoculated with BVG alone, or co-inoculated with BVG and CMoV, or BVG and tlaRNAs Gamma, Sigma, or ST9. Expected product size for BVG is 390 bp. Panel **(B)** shows products from RT-PCR based detection of CMoV, Gamma, Sigma, and ST9 in *N. benthamiana* plants co-inoculated with BVG and each of these viruses. Expected product sizes are as follows: CMoV=532 bp; Gamma=534 bp; Sigma=399 bp; ST9=430 bp. We suspect the faint bands in the gel image for Gamma detection are likely from minor cross contamination or nonspecific primer binding. RT+: reverse transcription positive control; PCR+: plasmids used as PCR positive controls – some of these did not amplify, we suspect too much plasmid was used in the reaction; NTC: no template control.
