# Supplementary figures and images for: Dissecting dynamic plant virus synergism in mixed infections of poleroviruses, umbraviruses, and tombusvirus-like associated RNAs
Source: Front Microbiol. 2023 Jul 6;14:1223265. doi: 10.3389/fmicb.2023.1223265 (PMC10359716; doi:10.3389/fmicb.2023.1223265)

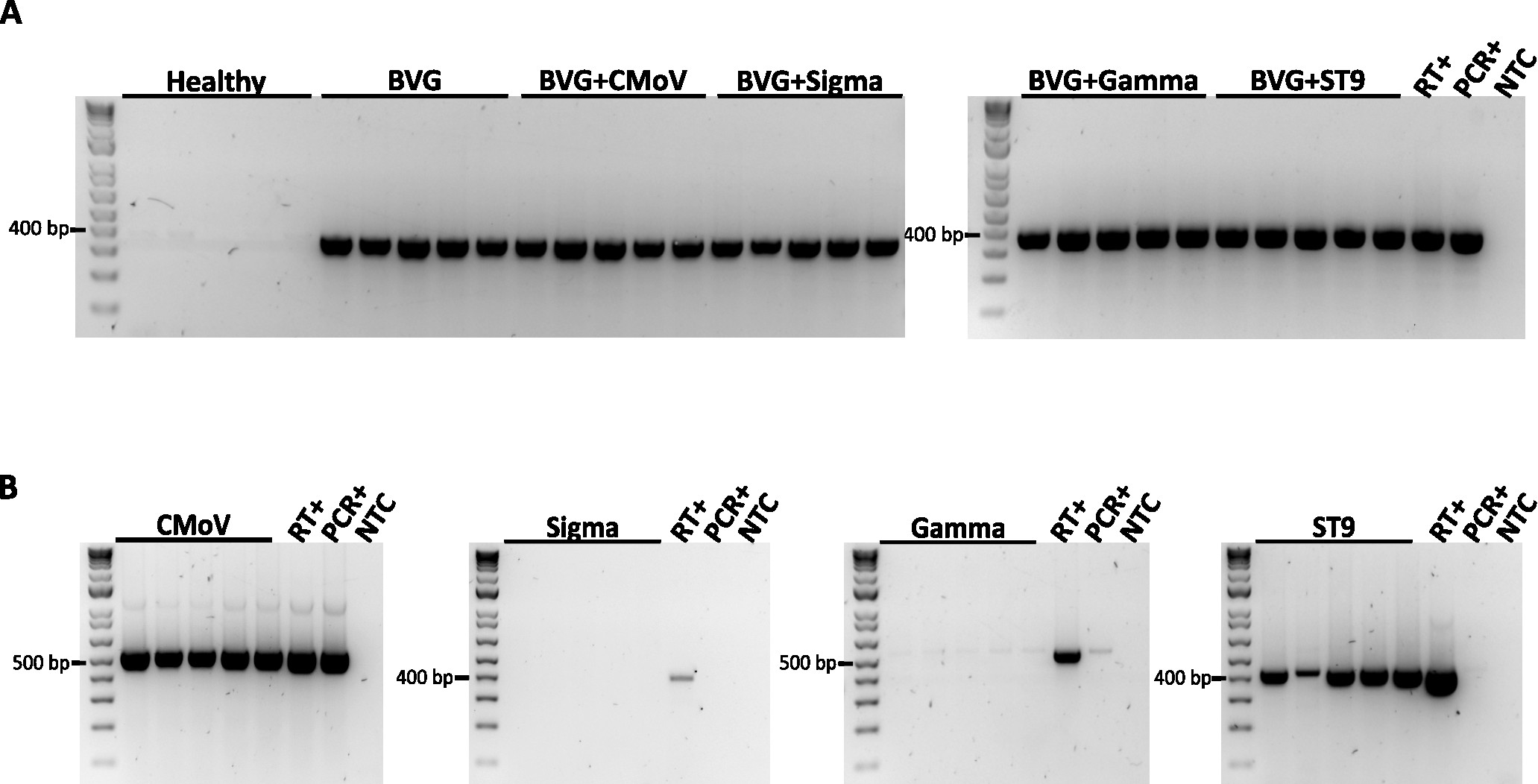

Supplement: SUPPLEMENTARY FIGURE S1 — RT-PCR data of from BVG + tlaRNA co-inoculation experiments. Panel (A) shows the products obtained from RT-PCR based detection of BVG in Nicotiana benthamiana plants that were agroinoculated with BVG alone, or co-inoculated with BVG and CMoV, or BVG and tlaRNAs Gamma, Sigma, or ST9. Expected product size for BVG is 390 bp. Panel (B) shows products from RT-PCR based detection of CMoV, Gamma, Sigma, and ST9 in N. benthamiana plants co-inoculated with BVG and each of these viruses. Expected product sizes are as follows: CMoV=532 bp; Gamma=534 bp; Sigma=399 bp; ST9=430 bp. We suspect the faint bands in the gel image for Gamma detection are likely from minor cross contamination or nonspecific primer binding. RT+: reverse transcription positive control; PCR+: plasmids used as PCR positive controls – some of these did not amplify, we suspect too much plasmid was used in the reaction; NTC: no template control. [file Image_1.jpg]
