# Supplementary material for: Dissecting dynamic plant virus synergism in mixed infections of poleroviruses, umbraviruses, and tombusvirus-like associated RNAs
Source: Front Microbiol. 2023 Jul 6;14:1223265. doi: 10.3389/fmicb.2023.1223265 (PMC10359716; doi:10.3389/fmicb.2023.1223265)
Supplement: Supplementary file 2 [file Table_1.DOCX]

| **Table S1: Primers used for multiplexed RT-qPCR detection and relative quantification of viruses used in this study** | | | |  |
| --- | --- | --- | --- | --- |
| **Target** | **Primer/probe name** | **Sequence** | **Fluorophore** |  |
|  |  |  |  |  |
| **TuYV** | TuYV_qF | GGAAGGACTGTTAGGCTGTAAA |  |  |
|  | TuYV_qR | TAACCCAGCCATCTCTCTCA |  |  |
|  | TuYV_qP | TGCTTTGCACTTTGCTAGGTTGGC | Fam |  |
| **CMoV** | CMoV_qF | GTTAATCACCCAGGAGAGGATG |  |  |
|  | CMoV_qR | CATACTGGGCAACTGGTATGT |  |  |
|  | CMoV_qP | TCTCTGTTGAGCATGAGCGTTGGT | Cy5 |  |
| **Gamma** | Gam_qF | GAGCATGTGGGTCTTCTAGTTT |  |  |
|  | Gam_qR | CTCCACCATCTGGTTTCATCTT |  |  |
|  | Gam_qP | TAGTGCGCTCAGCTCCACATCAAA | Texas red |  |
| **Sigma** | Sig_qF | ATGCAAGGAGGGCACATAC |  |  |
|  | Sig_qR | TCACAAACCACCCTCGTAATC |  |  |
|  | Sig_qP | TGTCACTATCGCCGGAACATCTGC | Texas red |  |
| **ST9** | ST9_qF | CGCATCTGGTTGAGGATAGTATAG |  |  |
|  | ST9_qR | GTAGACTGGACTCCCACAATTC |  |  |
|  | ST9_qP | AAACTGTGCTGGAGGTAGACGACC | Texas red |  |
| **Cytochrome C Oxidase** | Cox_qF | CGTCGCATTCCAGATTATCCA |  |  |
|  | Cox_qR | CAACTACGGATATATAAGRRCCRRAAC |  |  |
|  | Cox_qP | AGGGCATTCCATCCAGCGTAAGCA | Hex |  |
| Listed are the target viruses and corresponding primer names, sequences, and respective fluorophores used for the multiplexed RT-qPCR assay used in this study. | | | |  |
|  |  |  |  |  |
